# Influences of communication ability, organizational intimacy, and trust among colleagues on job satisfaction of nurses in comprehensive nursing care service units

**DOI:** 10.3389/fpubh.2024.1354972

**Published:** 2024-02-26

**Authors:** Sue Young Hahm, Minkyung Gu, Sohyune Sok

**Affiliations:** ^1^Department of Nursing, Graduate School, Kyung Hee University, Seoul, Republic of Korea; ^2^Department of Nursing, College of Health Science, Daejin University, Pocheon-si, Republic of Korea; ^3^College of Nursing Science, Kyung Hee University, Seoul, Republic of Korea

**Keywords:** nurse, communication, organizational intimacy, colleagues, job satisfaction

## Abstract

**Background:**

Communication abilities, organizational intimacy, trust among colleagues, and job satisfaction of nurses in comprehensive nursing care service units are emphasized more than any other ward, and research on this is necessary.

**Objective:**

The study was to examine the influences of communication ability, organizational intimacy, and trust among colleagues on the job satisfaction of nurses in the comprehensive nursing care service units.

**Methods:**

This study used a cross-sectional descriptive design. The participants were 155 nurses caring for patients in the comprehensive nursing care service units in Seoul. Measures included the general characteristics of study participants, communication ability, organizational intimacy, trust among colleagues, and job satisfaction. The data were analyzed using the SPSS/WIN version 27.0 program. The data collection period was from August to September 2022.

**Results:**

Factors influencing the job satisfaction of nurses at the comprehensive nursing care service units were organizational intimacy (*β* = 0.36), communication abilities (*β* = 0.26), trust among colleagues (*β* = 0.22), and the average number of patients assigned to a nurse (*β* = −0.19), which explained 67% of the variance.

**Conclusions:**

Organizational intimacy was the greatest factor influencing the job satisfaction of nurses in the comprehensive nursing care service units. To increase the job satisfaction of ward nurses working in the comprehensive nursing care service, securing manpower is required, and it is necessary to provide effective nursing care with an average number of patients of 5 or less. In particular, a systematic job training program is needed to increase organizational intimacy among team nurses.

## Introduction

A comprehensive nursing care service unit is a ward providing nursing care with integrated services necessary for properly placing nursing staff and patient safety management in an environment with facilities, equipment, etc., by forming a team consisting of only professional nursing staff without guardians or caregivers staying (1, 2). In South Korea, since 2015, comprehensive nursing care service units have been converted into a pilot project for applying for health insurance benefits and are being expanded to all hospitals, including tertiary general hospitals with more than 500 beds (1). In comprehensive nursing care service units, based on cooperation and coordination among nursing staff, nurses and nursing organization staff work as a team to provide continuous and holistic nursing care (1–3). Therefore, to efficiently operate an effective, comprehensive nursing care service unit and further strengthen the team's communication abilities beyond their nursing performance abilities, specific methods are required.

Communication abilities are important to provide nursing care among members of a comprehensive nursing care service unit. Effective communication abilities for nurses in the comprehensive nursing care service unit can significantly increase the therapeutic effect on patients and help nurses achieve nursing goals for their duties (3, 4). In particular, it may promote the performance and development of the nursing organization by reducing physical friction among members to a minimum and helping them obtain a sense of psychological stability (3, 5–7). On the other hand, since careless and unclear communication abilities of nurses can sometimes make outcomes of nursing negative and cause safety accidents to patients (8–11), it can inevitably act as a factor that can lower organizational intimacy among nurses and members of the nursing organization and trust among colleagues (6, 9, 11).

Organizational intimacy means that the relationship among people working together is very close and strong, and it encompasses all situations such as the desire to be together, solidarity, and building closeness (12, 13). Organizational intimacy within a nursing organization has been reported to increase immersion in the teamwork of team members, induce voluntary and positive behavior, increase customer orientation, and improve job satisfaction (14, 15). Above all, for nurses in the comprehensive nursing care service unit, working in shifts and division of work are done at the same time during limited hours due to the nature of their duties, so intimacy within the team acts as a major factor (9, 11).

On the other hand, to build intimacy within a team in a nursing organization, trust among colleagues who want to accept the other person's vulnerability based on positive expectations for team members is more important than anything else (12, 13, 15, 16). Trust among colleagues refers to respecting the words, actions, and decision-making of the other person on the premise of mutual trust among members of the nursing organization (17). This plays a decisive role in reducing turnover intentions among members of the nursing organization and increasing job satisfaction (16, 18, 19).

In the end, communication abilities, organizational intimacy, and trust among colleagues can act as antecedent variables that positively affect the job satisfaction of nurses (20–22). Thus, they should be further emphasized for nurses in comprehensive nursing care service units who perform nursing duties with the nursing staff. In other words, the institutionalization of comprehensive nursing care services has increased the time nurses provide direct nursing care to hospitalized patients, and as the needs of hospitalized patients have been met, the time nursing personnel spend together in a cooperative relationship has increased. It can be predicted that this may have a positive effect on the job satisfaction felt by nurses. Therefore, empirical research on this is of utmost importance.

Nevertheless, most studies related to nurses in comprehensive nursing care service units are limited to studies on nurses' job stress, role conflict, emotional labor, and nursing work roles, whereas there is a lack of studies identifying job satisfaction, communication abilities, organizational intimacy, and trust among colleagues in nurses in comprehensive nursing care service units (18, 23, 24). Therefore, identifying the levels of communication abilities, organizational intimacy, and trust among colleagues and the relationship between each variable to investigate the factors affecting the job satisfaction of nurses working in comprehensive nursing care service units is necessary.

This study will be able to suggest specific measures to improve the quality of nursing care for ward nurses working in comprehensive nursing care service units. Particularly, it will be able to provide fundamental data for the utilization of human resources for the effective operation of a team nursing system in comprehensive nursing care service units.

The purpose of this study was to examine the levels of communication ability, organizational intimacy, trust among colleagues, and job satisfaction of nurses in comprehensive nursing care service wards. Also, it was to examine and identify the factors influencing the levels of job satisfaction of nurses in comprehensive nursing care service wards. The specific aims of this study were to (1) identify the general characteristics of nurses in the comprehensive nursing care service unit; (2) examine the differences in job satisfaction, communication ability, organizational intimacy, and trust among colleagues according to the general characteristics of nurses in the comprehensive nursing care service unit; (3) examine the correlations between job satisfaction, communication ability, organizational intimacy, and trust among colleagues of nurses in the comprehensive nursing care service unit; and (4) examine the factors influencing the job satisfaction of nurses in the comprehensive nursing care service unit.

## Methods

### Study population

A cross-sectional descriptive design was used. The study participants were nurses who had been working for more than 6 months in the comprehensive nursing care service unit at two general hospitals in Seoul, South Korea. New nurses with <6 months of work experience after joining the hospital were excluded from the study as they were considered neophytes in performing nursing duties independently. The number of study participants was calculated with a significance level of 0.05 and a medium effect size of 0.15 to secure 80.0% of the statistical power for regression analysis using the G^*^Power 3.1.5 program (25). As a result, 139 study participants were determined to be the appropriate sample size, and the questionnaire was distributed to 160 subjects to fill out in consideration of the dropout rate. Of the 160 questionnaires, five copies were excluded as they were not returned; hence, 155 copies (96.88%) were retrieved and used for the study's statistical analysis.

### Measurements

#### Study participant's general characteristics survey

Based on a literature review and previous research, a set of general characteristics of study participants included gender, age, level of education, job position, total clinical career, current department career, type of work, average number of patients, and annual salary. This consisted of a total of nine items.

#### Communication ability

Communication abilities were measured with a communication ability assessment scale developed by Lee and Kim (4). This scale consists of 15 items, namely, self-exposure, understanding someone else's situation, social tension relief, assertiveness, concentration, interaction management, expressiveness, empowerment, immediacy, efficiency, social appropriateness, logic, goal understanding, responsiveness, and control. The items include “I allow my coworkers to know who I really am,” “I listen intently to what my coworkers say when I talk to them,” “I can easily recognize the purpose of my coworkers' conversation during a conversation,” and “I speak logically to the other person.” Each item was scored on a five-point Likert scale and ranged from 15 to 75 points. On this scale, the higher the score, the higher the communication abilities. The scale's reliability was Cronbach's α = 0.83 in Lee and Kim's study (4) and Cronbach's α = 0.93 in this study.

#### Organizational intimacy

Organizational intimacy was measured using a scale modified by Kim and Lee (13). This tool consists of 12 items divided into two categories, namely, opportunities for forming intimacy and expansion of intimacy. The items consist of contents such as “When a problem arises during work, I solve it together with my colleagues,” “I have the opportunity to form personal relationships with my colleagues at work,” “I have very close colleagues at work,” and “I am happy working with my colleagues.” Each item is scored on a five-point Likert scale, with higher scores indicating higher organizational intimacy. The scale's reliability was Cronbach's *?* = 0.79 in Kim and Lee's study (13) and Cronbach's *?* = 0.92 in this study.

#### Trust among colleagues

Trust among colleagues was measured using a scale modified by Baek (15). This scale includes 12 items divided into three sub-dimensions: four on competence, four on consideration, and four on sincerity. The items include contents such as “My teammates successfully complete their tasks,” “My teammates truly care about what is important to me,” “I try to treat my teammates with sincerity,” and “Teammates consistently take action.” Each item is scored on a five-point Likert scale, with higher scores indicating higher colleague trust. The scale's reliability was Cronbach's *?* = 0.95 in Baek's study (15) and Cronbach's *?* = 0.97 in this study.

#### Job satisfaction

Job satisfaction was measured using the Job Satisfaction Scale for Clinical Nurses (JSS-CN), developed by Lee et al. (16). The scale encompasses six sub-dimensions with 33 items: nine on organizational recognition and professional achievement, six on human maturity through the nursing profession, eight on respect and recognition of human relations, four on fulfillment of duties as a nurse, three on professional competency, and three on job stability and worthiness. The items consist of contents such as “I am demonstrating outstanding capabilities as a professional,” “I am grateful for my job as a nurse,” “I find my work as a nurse to be a source of vitality in my life,” and “The career of a nurse suits my personality.” Each item is scored on a five-point Likert scale, with higher scores indicating higher job satisfaction. The scale's reliability was Cronbach's *a* = 0.95 at the time developed by Lee et al. (16) and Cronbach's *?* = 0.97 in this study.

### Data collection

The duration of data collection was from June to August 2022. Researchers contacted prospective study participants and explained the purpose of this study, and the details of participation, and the questionnaires used. Researchers obtained written informed consent from study participants who agreed to participate in this study. The questionnaire was only provided to study participants who agreed to participate in the study. The survey consisted of a self-report questionnaire administered by the researcher. Each questionnaire took ~20–25 min to complete.

### Ethical considerations

This study proceeded with data collection after receiving approval from the Institutional Review Board of a hospital. The researcher visited the hospital nursing unit, explained the purpose of the study to each department head, and obtained consent. All subjects participating in this study were explained about the purpose of the study, the provision of personal information, and confidentiality related to anonymity. In addition, it was explained that they could withdraw from the study at any time if they wanted to stop participating in it.

### Data analysis

The SPSS PC+ version 27.0 statistical software program analyzed the collected data in this study. The general characteristics of the study participants were analyzed using frequency and percentage in descriptive statistics. The levels of study variables were analyzed using the mean and standard deviation in descriptive statistics. Differences in the levels of communication ability, organizational intimacy, trust among colleagues, and job satisfaction according to the general characteristics of the study participants were analyzed using a *t*-test, an ANOVA with Scheffe a *post-hoc* test. Correlations between job satisfaction among nurses and related factors were analyzed using Pearson's correlation coefficient. To examine the factors influencing the job satisfaction of nurses in comprehensive nursing care service wards, multiple regression analysis was used. The statistical significance criterion for the study results was a *p*-value of <0.05.

## Results

### General characteristics of the study participants

There were 155 subjects in this study, with 148 female (95.5%) and seven male (4.5%) subjects. The majority of the subjects (66 subjects or 42.6%) were 26–30 years old, and the average age was 30.23 years. Most subjects (111 subjects or 71.6%) graduated from a 4-year college, and 143 subjects (92.3%) were nurses. Then, the majority of the subjects (70 subjects or 45.2%) had clinical experience for more than 6 years, and the average clinical experience was 7.04 years. In addition, the majority of the subjects (80 subjects or 51.6%) had more than 3 years of experience in the comprehensive nursing care service unit, and the average career was 2.62 years. Moreover, most of the subjects (138 subjects or 89.0%) had three shifts, and 94 subjects (60.7%) were assigned to 6–10 patients. Finally, in terms of the annual salary of the subjects, it was 45 million KRW or more for 71 subjects (45.8%), which was the most common (Table 1).

**Table 1:** General characteristics of the study participants.

**Characteristics**	***n***	***%***	**Mean ±SD**
**Gender**			
Female	148	95.5	
Male	7	4.5	
**Age (year)**			
≤ 25	38	24.5	30.23 ± 6.81
26–30	66	42.6	
≥31	51	32.9	
**Level of education**			
College	20	12.9	
University	111	71.6	
≥Master	24	15.5	
**Job position**			
Staff nurse	143	92.3	
Charged nurse	12	7.7	
**Total clinical career (year)**			
< 3	41	26.4	7.04 ± 6.97
3–6	44	28.4	
>6	70	45.2	
**Current departmental career (year)**			
≤ 1	31	20.0	2.62 ± 1.82
2–3	44	28.4	
>3	80	51.6	
**Type of work**			
3 shifts	138	89.0	
2 shifts	9	5.8	
N keep	8	5.2	
**Average number of patients**			
≤ 5	9	5.8	
6–10	94	60.7	
11–15	52	33.5	
**Annual salary (10,000 won)**			
< 4,000	32	20.7	
4,000–4,500	52	33.5	
>4,500	71	45.8	

### Levels of job satisfaction, communication ability, organizational intimacy, and trust among colleagues

The range of job satisfaction scores of the study participants was 33–165 points, with a median of 115 points and an average of 113.00 points. The range of communication ability scores was 15–75 points, with a median of 56 points and an average of 54.76 points. The range of organizational intimacy scores was 12–60 points, with a median of 44 points and an average of 42.57 points. The range of trust scores among colleagues was 12–60 points, with a median of 45 points and an average of 42.96 points. The levels of the study participants' job satisfaction, communication ability, organizational intimacy, and trust among colleagues were slightly lower than the median values.

### Differences in job satisfaction, communication ability, organizational intimacy, and trust among colleagues according to the general characteristics of study participants

In this study, job satisfaction according to general characteristics showed a statistically significant difference in age (*F* = 3.48, *p* = 0.033), the level of education (*F* = 7.60, *p* = 0.001), job position (*t* = −2.38, *p* = 0.019), type of work (*F* = 4.38, *p* = 0.014), the average number of patients assigned to the nurse (*F* = 7.88, *p* = 0.001), and annual salary (*F* = 9.56, *p* = 0.001). As a result of the *post-hoc* test, job satisfaction was higher in “25 years or younger” and “31 years or older” than “26–30 years” old subjects, in “junior college graduate” and “graduate school or higher” than “4-year collage graduate” for the level of education, in “2 shifts” than “N keep” for the type of work, in “5 or fewer patients” than “6–10 patients” and “11–15 patients” for the average number of patients assigned for the nurse, and in “ <40 million KRW” and “45 million KRW or more” than “40–45 million KRW” for the annual income. Next, communication abilities, according to general characteristics, showed a statistically significant difference in the type of work (*F* = 7.15, *p* = 0.001), the average number of patients assigned to the nurse (*F* = 3.30, *p* = 0.040), and annual salary (*F* = 11.57, *p* = 0.001). As a result of the *post-hoc* test, the level of communication abilities was higher in “3 shifts” and “2 shifts” than “N keep” for the type of work, in “5 or less patients” than “6–10 patients” and “11–15 patients” for the average number of patients assigned to the nurse, and in “ <40 million KRW” and “45 million KRW or more” than “40–45 million KRW” for the annual income. Organizational intimacy, according to general characteristics, showed a statistically significant difference in age (*F* = 4.22, *p* = 0.016), type of work (*F* = 4.11, *p* = 0.018), and annual salary (*F* = 5.94, *p* = 0.003). As a result of the *post-hoc* test, organizational intimacy was higher in “25 years or younger” and “31 years or older” than “26–30 years” for the age, in “3 shifts” and “2 shifts” than “N keep” for the type of work, and in “ <40 million KRW” and “45 million KRW or more” than “40–45 million KRW” for the annual income. Finally, trust among colleagues according to general characteristics showed a statistically significant difference in annual salary (*F* = 7.84, *p* = 0.001). As a result of the *post-hoc* test, trust among colleagues was higher in the annual salary of “ <40 million KRW” and “45 million KRW or more” than “40–45 million KRW” (Table 2).

**Table 2:** Differences in job satisfaction, communication ability, organizational intimacy, and trust among colleagues according to general characteristics of study participants.

**Job satisfaction**			**Communication ability**		**Organizational intimacy**		**Trust among colleagues**	
**Characteristics**	**Mean** ±**SD**	***t*** **or** ***F*****(*****p*****)** ***Scheffe***	**Mean** ±**SD**	***t*** **or** ***F*****(*****p*****)** ***Scheffe***	**Mean** ±**SD**	***t*** **or** ***F*****(*****p*****)** ***Scheffe***	**Mean** ±**SD**	***t*** **or** ***F*****(*****p*****)** ***Scheffe***
**Gender**								
Female	113.31 ± 22.50	0.79 (0.432)	54.81 ± 9.23	0.31 (0.760)	42.64 ± 8.04	0.48 (0.635)	43.01 ± 9.68	0.31 (0.758)
Male	106.43 ± 25.58		53.71 ± 10.00		41.14 ± 9.46		41.86 ± 9.65	
**Age (year)**								
≤ 25^a^	113.24 ± 18.36	3.48 (0.033^*^) a, c > b	56.21 ± 8.08	2.40 (0.095)	44.63 ± 7.16	4.22 (0.016^*^)a, c > b	44.92 ± 8.87	2.92 (0.057)
26–30^b^	108.15 ± 24.67		52.89 ± 10.24		40.45 ± 8.87		40.83 ± 10.41	
≥31^c^	119.10 ± 21.55		56.10 ± 8.37		43.76 ± 7.11		44.25 ± 8.79	
**Level of education**								
College^a^	121.50 ± 16.99	7.60 (0.001^*^) a, c > b	56.95 ± 5.35	1.99 (0.141)	46.00 ± 5.73	2.74 (0.068)	44.35 ± 8.44	0.24 (0.785)
University^b^	108.77 ± 21.82		53.84 ± 9.93		41.70 ± 8.36		42.71 ± 9.80	
≥Master^c^	125.46 ± 24.38		57.21 ± 7.85		43.71 ± 7.81		42.96 ± 10.18	
**Job position**								
Staff nurse	111.77 ± 22.43	−2.38 (0.019^*^)	54.39 ± 9.27	−1.73 (0.085)	42.28 ± 8.20	−1.54 (0.126)	42.94 ± 9.74	−0.11 (0.914)
Charged nurse	127.67 ± 20.10		59.17 ± 7.87		46.00 ± 5.72		43.25 ± 8.82	
**Total clinical career (year)**								
< 3^a^	109.61 ± 20.63	1.32 (0.271)	54.15 ± 10.44	0.86 (0.426)	42.80 ± 9.34	0.30 (0.743)	43.88 ± 10.58	0.67 (0.516)
3–6^b^	111.09 ± 22.81		53.66 ± 9.33		41.77 ± 7.75		41.59 ± 8.58	
>6^c^	116.19 ± 23.45		55.81 ± 8.41		42.93 ± 7.57		43.29 ± 9.76	
**Current department career (year)**								
≤ 1^a^	116.10 ± 19.08	0.49 (0.617)	56.35 ± 6.55	1.12 (0.330)	44.26 ± 7.29	1.41 (0.247)	4.65 ± 9.39	0.61 (0.545)
2–3^b^	110.86 ± 22.50		53.20 ± 11.47		41.11 ± 9.07		42.30 ± 10.30	
>3^c^	112.97 ± 24.00		55.00 ± 8.71		42.71 ± 7.76		42.68 ± 9.43	
**Type of work**								
3 shifts^a^	113.28 ± 22.19	4.38 (0.014^*^) b > c	55.09 ± 8.58	7.15 (0.001^*^) a, b > c	42.80 ± 7.68	4.11 (0.018^*^) a, b > c	43.35 ± 9.22	2.40 (0.094)
2 shifts^b^	125.56 ± 22.58		59.33 ± 7.92		45.56 ± 6.52		43.44 ± 9.25	
N keep^c^	94.13 ± 20.29		44.00 ± 14.25		35.25 ± 12.75		35.75 ± 14.98	
**Average number of patients**								
≤ 5^a^	135.33 ± 22.98	7.88 (0.001^*^) a > b, c	62.22 ± 6.02	3.30 (0.040^*^) a > b, c	44.00 ± 6.19	0.28 (0.758)	46.89 ± 10.14	1.20 (0.303)
6–10^b^	114.77 ± 22.17		54.53 ± 9.81		42.73 ± 8.25		43.26 ± 9.83	
11–15^c^	105.94 ± 20.46		53.88 ± 8.11		42.02 ± 8.16		41.75 ± 9.18	
**Annual salary (10,000 won)**								
< 4,000^a^	117.37 ± 18.73	9.56 (0.001^*^) a, c > b	58.34 ± 7.82	11.57 (0.001^*^) a, c > b	44.31 ± 6.49	5.94 (0.003^*^) a, c > b	45.31 ± 7.50	7.84 (0.001^*^) a, c > b
4,000–4,500^b^	102.44 ± 23.34		50.15 ± 10.90		39.52 ± 10.20		38.83 ± 11.42	
>4,500^c^	118.76 ± 21.13		56.52 ± 7.02		44.01 ± 6.24		44.93 ± 8.11	

^*^p < 0.05. a, b, c mean Scheffe *post hoc* test.

### Correlations between job satisfaction and study variables

The correlations between job satisfaction and study variables were communication ability (γ = 0.63, *p* < 0.01), organizational intimacy (γ = 0.65, *p* < 0.01), and trust among colleagues (γ = 0.61, *p* < 0.01), and they were found to be positively correlated (Table 3).

**Table 3:** Correlations between job satisfaction and factors related to it.

**Variables**	**Job satisfaction**	**Communication ability**	**Organizational intimacy**	**Trust among colleagues**
	***r(p)***			
Job satisfaction	1	0.73^*^	0.75^*^	0.71^*^
Recognition from the organization and professional achievement	0.94^*^	0.60^*^	0.67^*^	0.62^*^
Personal maturation through the nursing profession	0.93^*^	0.64^*^	0.60^*^	0.57^*^
Interpersonal interaction with respect and recognition	0.90^*^	0.73^*^	0.78^*^	0.74^*^
Accomplishment of accountability as a nurse	0.83^*^	0.68^*^	0.60^*^	0.56^*^
Display of professional competency	0.85^*^	0.69^*^	0.74^*^	0.68^*^
Stability and job worth	0.65^*^	0.48^*^	0.42^*^	0.42^*^
Communication ability	0.63^*^	1	0.77^*^	0.69^*^
Organizational intimacy	0.65^*^	0.77^*^	1	0.78^*^
Trust among colleagues	0.61^*^	0.69^*^	0.78^*^	1
Competence	0.65^*^	0.64^*^	0.71^*^	0.94^*^
Consideration	0.69^*^	0.61^*^	0.73^*^	0.93^*^
Sincerity	0.64^*^	0.68^*^	0.76^*^	0.94^*^

^*^p < 0.01.

### Factors influencing job satisfaction

The regression model's assumptions were tested. First, as a result of looking at the residual plot, homogeneity of variance was confirmed. Then, after testing the autocorrelation of errors with Durbin Watson to verify the independence of the residuals, the statistic was 1.68, and the test statistic was between 1.59 and 1.76, indicating that the assumptions of the regression equation were all satisfied without autocorrelation. In addition, the tolerance of multicollinearity was 0.28–0.91, which was >0.10, and the variance inflation factor (VIF) was 1.13–3.94, which was not >10, so all variables in this study showed no multicollinearity problem. In the analysis of the influencing factors for the subjects' job satisfaction, the regression model including general characteristics was statistically significant (*F* = 26.59, *p* < 0.001). Statistically significant variables appeared in the order of organizational intimacy (*β* = 0.36, *p* < 0.001), communication abilities (*β* = 0.26, *p* = 0.001), trust among colleagues (*β* = 0.22, *p* = 0.007), and the average number of patients assigned to a nurse (*β* = −0.19, *p* < 0.001). The explanatory power of the final regression model was 67.0% (Table 4).

**Table 4:** Factors influencing the job satisfaction.

**Variables**	***B***	**SE**	***β***	***t***	***p*-value**
Gender	−1.25	5.38	−0.01	−0.23	0.817
Age (year)	3.20	2.69	0.11	1.19	0.235
Level of education	0.91	2.19	0.02	0.42	0.679
Job position	2.23	4.81	0.03	0.46	0.643
Total clinical career (year)	0.73	2.52	0.03	0.29	0.774
Current departmental career (year)	−2.05	1.65	−0.07	−1.25	0.214
Type of work	−2.79	2.51	−0.06	−1.11	0.268
Average number of patients	−7.63	2.09	−0.19	−3.66	< 0.001^*^
Annual salary (10,000 won)	−0.86	1.98	−0.03	−0.432	0.667
Communication ability	0.63	0.19	0.26	3.26	0.001^*^
Organizational intimacy	0.99	0.25	0.36	3.93	< 0.001^*^
Trust among colleagues	0.51	0.19	0.22	2.73	0.007^*^
Adj *R*^2^ = 0.67, *F* = 26.59, *p* < 0.001^*^					

Dummy variable = 2 shifts, N keep.

Adj R^2^, adjust R-squared; SE, standard error.

^*^p < 0.05.

## Discussion

The levels of job satisfaction, communication abilities, organizational intimacy, and trust among colleagues of nurses in comprehensive nursing care service units were similar to the average values in most cases, which was consistent with the studies of Lee and Kim (5) and Lee and Jung (21) conducted for nurses in comprehensive nursing care service units. Through a study on the professional self-concept, work environment, and organizational intimacy of nurses in comprehensive nursing care service units, Lee and Jung (21) mentioned that communication strategies and techniques are needed to increase organizational intimacy. This will eventually improve the quality of nursing continuously and generally by teaming up with nursing organization personnel due to the nature of their work (11, 17–19). Therefore, to improve the job satisfaction of nurses in comprehensive nursing care service units, increase trust among colleagues, and improve working relationships, efforts such as developing various programs related to nurses' communication abilities and using continuing education should be made (23, 26).

Next, in the verification of differences in job satisfaction, communication abilities, organizational intimacy, and trust among colleagues according to the general characteristics of nurses in comprehensive nursing care service units, those aged 25 years or younger or 31 years or older had higher job satisfaction and organizational intimacy than those aged 26–30 years. This indicates that mid-career nurses had lower job satisfaction and organizational intimacy than novice or experienced nurses. It might result in mid-career nurses who have had some experience expressing critical opinions about conflict situations and the organization (24, 27). In addition, the smaller the average number of patients assigned to the nurse, the higher the job satisfaction and communication abilities, a similar result to the studies of Hao et al. (28) and Park et al. (29). Thus, medical institutions need to arrange nursing staff according to the number of patients in the comprehensive nursing care service unit in the future. The nurses with lower or higher annual salaries had higher job satisfaction, communication abilities, organizational intimacy, and trust among colleagues. This result seems to go along with the results of lower job satisfaction and organizational intimacy in mid-career nurses than in novice or experienced nurses. Therefore, it is necessary to improve treatment such as promotion opportunities and welfare benefits such as salary increases at the nursing organization level through an appropriate compensation system based on the nurses' work in comprehensive nursing care service units (8). In addition, for mid-career nurses in comprehensive nursing care service units, it is necessary to improve the nursing environment with continuous interest and effort by operating various programs such as education and activities that can strengthen the relationship with members of the nursing organization, as well as free and convenient ways to express their opinions about the nursing organization.

In this study, it was found that there were positive correlations between job satisfaction and communication abilities, organizational intimacy, and trust among colleagues of nurses in comprehensive nursing care service units. These results align with the studies of Lee and Gu (9), and Baek (15), which found that nurses' communication abilities, organizational intimacy, and trust among colleagues positively affected job satisfaction. To operate team nursing efficiently and increase the job satisfaction of nurses in comprehensive nursing care service units, an atmosphere should be created, and securing opportunities for cooperation, resting space, etc. should be emphasized (18, 19, 30). Moreover, to restore intimacy within the team and a culture of trust among colleagues, removing obstacles that hinder trust within the organization and striving to build a trustworthy culture between organizations are essential (31).

Finally, in this study, the factors affecting the job satisfaction of nurses in comprehensive nursing care service units were in the order of organizational intimacy, communication abilities, trust among colleagues, and the average number of patients assigned to the nurse. It is difficult to make a direct comparison as there are not many previous studies on the job satisfaction of nurses in comprehensive nursing care service units for medical institutions in South Korea. However, this study supports the results of previous studies by Han et al. (17), Kim et al. (19), and Park and Cho (22), concluding that organizational intimacy with the nursing organization staff and other medical personnel leads to effective communication, thereby further increasing the therapeutic effects for patients (7, 11, 32, 33). Therefore, to increase the job satisfaction of nurses in comprehensive nursing care service units, smooth and quick manpower securing is required, and an appropriate number of patients, an average of five or fewer, should be assigned to perform effective nursing care (1, 8). To increase the job satisfaction of nurses in comprehensive nursing care service units, personal management is needed to identify individual job suitability, such as by providing regular interviews to new nurses and younger nurses who have lower job satisfaction than experienced nurses (23). Additionally, it is necessary to develop, actively recommend, and operate various programs that can positively increase organizational intimacy and trust among nurses' colleagues in comprehensive nursing care service units (9, 11, 34).

## Implications for practice, policy, and research

It is necessary to develop various programs that can improve the communication abilities of medical institutions' nurses to increase their job satisfaction, especially the nurses who provide comprehensive nursing care services. The results of this study can be used to establish a theoretical framework for efficient intervention research to consistently and stably operate comprehensive nursing care service units. The important consideration in institutionalizing comprehensive nursing care services is patient safety and the provision of quality nursing services, and for this, it is important for nurses to be satisfied with their job roles. Moreover, to increase the job satisfaction of nurses working in comprehensive nursing care service units, securing manpower is required, and effective nursing care should be provided with an appropriate number of patients (e.g., an average of five or fewer). Ultimately, this will improve the quality of nursing care in comprehensive nursing care service units. The implications for nursing policy suggest that organizational intimacy, communication abilities, trust among colleagues, and the average number of patients assigned to the nurse should be considered for improving the job satisfaction of nurses in comprehensive nursing care service units. Also, how can the average number of patients (e.g., an average of five or fewer) specifically affect job satisfaction, and how much competency is needed for nurses in each department compared to the average number of patients? There is a need to verify this in more detail. Based on the results of this study, a program that can increase organizational intimacy and trust among colleagues of nurses in comprehensive nursing care service units that reflect the characteristics of Korean society may be designed in the future by expanding the factors affecting the job satisfaction of nurses in comprehensive nursing care service units. Conducting an experimental study is also recommended to verify its feasibility.

## Limitations and future directions

Research on nurses in comprehensive nursing care service units was insufficient, and the subjects of this study were nurses in comprehensive nursing care service units of two general hospitals located in Seoul. Thus, there are limitations in explaining the factors affecting nurses' job satisfaction in comprehensive nursing care service units due to the limited sample size. Therefore, it is necessary to conduct replication and expansion studies that will consider more extensive subject sampling. Based on this study, it is necessary to identify a practical team nursing system for nurses working in comprehensive nursing care service units and to correct and improve problems. At the same time, more active interest and efforts from the government and medical institutions are needed to stably establish this system in Korean society.

## Conclusion

The factors affecting the job satisfaction of nurses in comprehensive nursing care service units were organizational intimacy, communication abilities, trust among colleagues, and the average number of patients assigned to the nurse. This study is meaningful since it highlighted the need for various programs that can increase communication abilities, organizational intimacy, and trust among colleagues for the job satisfaction of nurses in comprehensive nursing care service units and presented fundamental data to improve the job satisfaction of nurses in comprehensive nursing care service units.

## Data availability statement

The raw data supporting the conclusions of this article will be made available by the authors, without undue reservation.

## Ethics statement

The studies involving humans were approved by Kangdong Sungsim Hospital Institutional Review Board (IRB No. 2022-05-002-002). The studies were conducted in accordance with the local legislation and institutional requirements. The participants provided their written informed consent to participate in this study.

## Author contributions

SH: Conceptualization, Data curation, Formal analysis, Investigation, Methodology, Validation, Visualization, Writing – original draft, Writing – review & editing. MG: Conceptualization, Formal analysis, Investigation, Methodology, Validation, Visualization, Writing – review & editing. SS: Conceptualization, Data curation, Formal analysis, Investigation, Methodology, Project administration, Resources, Software, Supervision, Validation, Visualization, Writing – original draft, Writing – review & editing.
